# Swimming Against the Current: Addressing Community Needs and Establishing Partnerships for the Prevention of Opioid and Methamphetamine use Among Parents

**DOI:** 10.1007/s11121-023-01602-5

**Published:** 2023-10-26

**Authors:** Ryan R. Singh, Jeffrey M. Peterson, Jason Chapman, Lisa Saldana

**Affiliations:** Chestnut Health Systems, Lighthouse Institute – OR, Group1255 Pearl St., Eugene, OR 97401 USA

**Keywords:** Parental opioid and/or methamphetamine, Prevention and treatment, Implementation, FAIR

## Abstract

The opioid and methamphetamine crises in Oregon have a consequential impact on young families, as an increasing number of parents experience substance use disorder (SUD). As parental substance use escalates, the child welfare system (CWS) becomes overwrought with families who have complex needs. The burden placed on families and on the CWS indicates a need for prevention and treatment interventions for parental SUDs. In response to the worst statewide opioid and methamphetamine epidemics in the USA, a Hybrid Type 2 trial of PRE-FAIR—a prevention intervention for parents—is being implemented in five Oregon counties. Establishing strong partnerships within the communities helped identify the need to implement the evidence-based FAIR treatment model alongside PRE-FAIR. A strong focus on implementation includes meeting the needs of communities and establishing the infrastructure necessary for sustainment of the FAIR programs at the provider agencies. Despite implementation efforts to direct toward PRE-FAIR referrals, parents in need of treatment are being referred at a disproportionate rate, as are older parents who fall outside of study-eligibility. Both challenges have delayed the ability to enroll a sufficient number of participants for the prevention trial. This commentary describes the impact of the opioid and methamphetamine epidemics in Oregon as the crises relate to implementing prevention versus treatment interventions—highlighting the importance of addressing community needs and establishing strong partnerships, which has allowed creative strategies to increase PRE-FAIR recruitment.

Since 2021, Oregon has ranked first in the nation for population-reported, prior year opioid and methamphetamine use (USDHHS, 2021). Together, the “twin” epidemics of opioid misuse and methamphetamine use (Ellis et al., 2018) have strained the child welfare system (CWS), as a growing number of families become involved due to parents presenting with opioid and/or methamphetamine use disorder (OUD; MUD). The public health burden of parental OUD/MUD has been exacerbated by two recent events. First, the impact of COVID-19 has catalyzed parental substance use initiation and escalation, as parents experienced increases in adverse mental health and reduced access to services (Walters et al., 2022). Second, the increasing prevalence of synthetic opioids (e.g., fentanyl) has given rise to a growing number of overdose and overdose-related deaths (Hedegaard et al., 2021), placing parents with OUD/MUD, and their families, at heightened risk. These events underscore the urgent need to implement services—both prevention and treatment—to support families across the state and to alleviate strain on the CWS.

The Families Actively Improving Relationships (FAIR) program is an intensive outpatient treatment intervention to support families involved with the CWS with active parental OUD/MUD. FAIR targets parental substance use, mental health, parenting skills, and ancillary social determinant of health needs. FAIR services and program maintenance rely on Medicaid reimbursement, which has enabled its sustainment in one Oregon county for over a decade. The demonstrated outcomes include decreased opioid and methamphetamine use, improvements in mental health, decreases in parental stress, and increased parental stability (Saldana et al., 2021).

In response to national efforts to intervene on the opioid and methamphetamine crises earlier in the prevention continuum, and to reduce the risk of family CWS involvement due to OUD/MUD, an adaptation of the FAIR model was made—PRE-FAIR. As an upstream prevention model, PRE-FAIR maintains the core components of FAIR, with less emphasis on substance use treatment. PRE-FAIR incorporates the same level of engagement (i.e., frequent, consistent meetings; use of incentives; home and community-based delivery) that is crucial to the ongoing effectiveness of FAIR (Cruden et al., 2021). Together, PRE-FAIR and FAIR can provide prevention and treatment intervention opportunities to communities in need.

## Community-based Implementation of PRE-FAIR

PRE-FAIR is being implemented as part of the NIDA-funded Helping to End Addiction Long-Term (HEAL) initiative, which focuses on prevention of opioid misuse among young adults. The randomized control trial employs a Hybrid Type 2 design to evaluate effectiveness and implementation in community-based practice settings. Aligning with the NIDA Request for Applications, the PRE-FAIR trial aimed to include young parents (age 16–30) who are insured through Medicaid. A year was spent developing a collaborative partnership with the Oregon Department of Human Services (ODHS) Child Welfare and Self-Sufficiency (CW/SS) program leadership to identify regions throughout the state in greatest need of preventive services. Rural regions were selected based on the risk for an open child welfare case due to factors which include parental OUD/MUD and the level of services in those regions for parents to access. Establishing this state-level partnership helped develop regional-level ODHS relationships, which led to identifying unmet needs regarding substance use treatment in these communities.

Working with ODHS, it was determined that, in addition to implementing PRE-FAIR, communities would benefit from the implementation of the FAIR treatment model at each provider agency. Implementing both programs gives communities access to new prevention and treatment options for parents in need. Using a standardized implementation process, operationalized using the Stages of Implementation Completion (SIC; Singh & Saldana, 2022), an implementation roadmap was developed for each community. The SIC defines implementation strategies/activities within eight standardized stages of implementation including (1) Engagement, (2) Consideration of Feasibility, (3) Readiness Planning, (4) Hiring and Training, (5) Establishing Fidelity Monitoring, (6) Program Launch, (7) Ongoing Quality Assurance Monitoring, and (8) Competency in program delivery. Activities target specific organizational skills, tasks, protocols, or efficiencies that need to be in place to build the necessary infrastructure for successful implementation (e.g., confirming the referral process with community partners in Readiness Planning). SIC activities for implementing the continuum of FAIR services—PRE-FAIR to FAIR—did not differ. Training providers in both programs have benefitted the provider agencies, as these agencies have a greater opportunity to maintain steady caseloads for sufficient reimbursement and can ensure that clinicians are consistently delivering the FAIR programs (i.e., PRE-FAIR and FAIR), allowing for increased opportunities to enhance the skills necessary to achieve program fidelity.

The implementation of PRE-FAIR includes a specific focus on long-term program sustainment. Therefore, implementation strategies are utilized to support study sites to gain the skills necessary to sustain PRE-FAIR after the study has concluded. Partnering with provider agencies in the earliest stages of implementation to establish a strong community coalition and begin conducting outreach to community partners has been key to consistent referral flows. Despite these efforts emphasizing the study focus on PRE-FAIR as a means of preventing the onset of parental OUD/MUD, challenges remain in conducting the clinical effectiveness part of the Hybrid 2 research trial in the context of Oregon’s staggering opioid and methamphetamine epidemics. That is, referrals are sufficient to the programs for implementation goals, but at the time of this writing, PRE-FAIR referrals, specifically, are insufficient to test the effectiveness of PRE-FAIR. Below, two recruitment challenges are described, both of which represent the need to balance what is necessary to effectively evaluate PRE-FAIR and its implementation while ensuring that the needs of the communities are addressed.

## Challenge 1: The Majority of Referrals Require Substance use Treatment

**Fig. 1:**
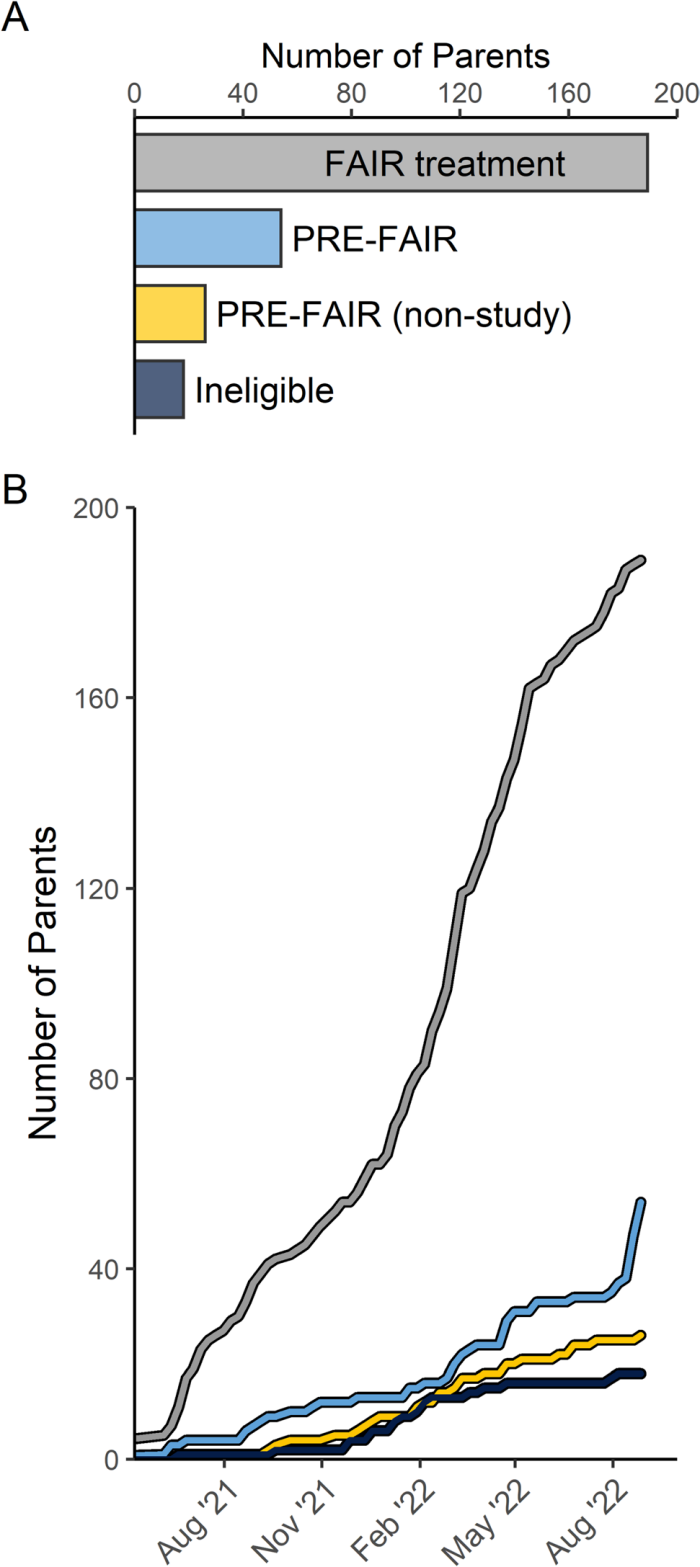
Distribution of parents screened and referral timeline

Consistent with the escalating rates of opioid misuse and methamphetamine use throughout Oregon, the majority of parents referred were screened out due to existing OUD/MUD. For the first year of the study period, parents with OUD/MUD were being referred at a rate of six to one compared to study-eligible upstream prevention participants. A recent jump in study-eligible referrals has reduced the rate to approximately four to one (Fig. 1A). Despite this reduction, treatment referrals continue to far outweigh those for prevention. The highly skewed number of referrals that require substance use treatment demonstrate the critical need for treatment services in the study regions and is indicative of the seriousness of the opioid and methamphetamine crises in Oregon. Further, the amount of attention that parents with active OUD/MUD demand from the CW/SS workforce likely creates a barrier to making referrals for parents who might benefit from PRE-FAIR. Evidence of this strain on the workforce is demonstrated by the large number of treatment referrals that are made directly by CW/SS workers, compared with the majority of study-eligible self-referrals from parents who have been encouraged by a CW/SS worker to get screened for the PRE-FAIR study themselves. If CW/SS workers are relying on parents in need of prevention to self-refer, it is possible that parents who could benefit from prevention services face obstacles to referring on their own.

Furthering the strain on the CW/SS workforce, the COVID-19 pandemic has intensified substance use, with the heaviest burden felt in rural communities (Walters et al., 2022). Risk factors that contribute to initiation or escalation of substance use include increased social isolation, compromised mental health, and housing insecurity (Walters et al., 2022). Further, the lack of effective support services for parents during the pandemic can exacerbate the opioid and methamphetamine crises (National Academies of Sciences, Engineering, and Medicine, 2021). The rural study recruitment location might intensify the effort needed to support parents impacted by COVID-19 and leave other parents who do not have active substance use concerns (but are at high risk) with less support.

## Challenge 2: Many Parents in Need of Prevention are Older

The high need for parental substance use treatment demonstrates the same level of need for prevention for parents. There have been a significant number of referrals for parents who do not have active OUD/MUD, are considered to have an elevated risk for substance use initiation or escalation, and yet are too old to meet eligibility for the PRE-FAIR study. In fact, nearly half of the referred parents who could benefit from substance use prevention are older than the eligible age range of 16 to 30 years old. Recent data shows the average age of first birth for both mothers and fathers has steadily increased in the USA (Mathews & Hamilton, 2016), and the highest rates of unintentional overdoses in Oregon occur between the ages of 35 and 54 for females, and 25 and 54 for males (Oregon Health Authority, 2022). Thus, as the epidemics continue, and with the rise in the use of synthetic opioids, parents with young children become particularly vulnerable to overdose. The older age range of unintentional overdose, particularly for females, suggests that efforts to support parents of all ages through prevention efforts are warranted as women become more likely to have young children at older ages. This challenge highlights a potential missed opportunity to study a population of parents in need of such prevention.

In response to Challenge 2, the study team worked with provider agencies and community partners to begin offering PRE-FAIR as a service to parents who fall outside the eligible age range, without inclusion in the study. Through offering a PRE-FAIR, non-study option, a substantial number of parents are being served with a goal of preventing parental onset of OUD/MUD (Fig. 1B).

## Strategies to Strengthen Recruitment While Ensuring Successful Implementation

A number of strategies to strengthen study recruitment were employed to address the two challenges described above. This response followed the success of focusing on implementation—leveraging strong partnerships and introducing the FAIR programs to communities. These strategies include the following:

1. Consistent Engagement with CW/SS Staff. Central to the recruitment efforts, is the extensive level of engagement with community partners. In collaboration with provider agency teams, early engagement with the CW/SS workforce was helpful in providing an introduction to FAIR in these communities, yet study-eligible referrals were not consistent until check-ins with CW/SS staff began to occur. There was an uptick in referrals as recruitment staff began attending routine staff meetings, to provide recommendations for eligible referrals, to remind workers and answer questions about PRE-FAIR, and to share success stories of parents involved with PRE-FAIR. This level of engagement has been necessary, and is consistent with other research related to the implementation of substance use prevention interventions (Substance Abuse and Mental Health Services Administration, 2022). Ongoing engagement over the course of implementation, particularly to demonstrate to community partners that there is tremendous benefit to upstream prevention for families, prior to onset of parental OUD/MUD, is warranted and possibly relevant to the implementation of other substance use prevention interventions.
2. Broaden Recruitment Efforts to Additional Community Partners. Fostering partnerships with groups within the legal and justice systems that work with parents who are in need of prevention services, schools and early childhood programs, and community-based organizations that work with parents (e.g., food banks, community centers) has opened the door to working with community partners beyond ODHS. Consistent engagement with these new partners provides an additional avenue to reach parents in need of prevention.
3. Tailored Recruitment Materials. To promote PRE-FAIR to ODHS and other community partners, a number of different recruitment materials were developed, including paper and digital brochures and flyers. Materials were tailored to each community or specific groups within communities (e.g., school districts). Monthly newsletters are sent to CW/SS staff, providing concise information about PRE-FAIR to the workforce.
4. Promote Self-Referrals. To promote self-referrals, flyers are placed in areas identified as having the greatest potential to be seen by study-eligible parents (e.g., food banks, laundromats, public libraries, health centers, shelters for the unhoused, childcare centers). All materials have been updated to include QR codes which, when scanned, direct parents to a brief survey to request to be screened for the study. Advertisements on social media sites, such as Facebook and Instagram, include information on how PRE-FAIR can help support parents, useful parenting tips, and tailored messages regarding local resources. The use of creative messaging on social media platforms has been a key strategy to reaching young parents directly. Like the paper-based materials, the social media advertisements include a link to a brief survey for parents. These simplified materials provide opportunities for early engagement with potential participants, allowing for parents to connect directly to someone who can explain PRE-FAIR in more detail and give parents a chance to ask questions and voice concerns. Thus, having a presence on social media platforms has been demonstrated in this project to reach a high volume of youth and young adults who might benefit from upstream prevention interventions.
5. Efficient Screening Process. Partnering with FAIR supervisors at each provider agency, an efficient process for the transfer of information from the referral and screening process to the request for services (PRE-FAIR or FAIR treatment) at the provider agency was developed. The goal is for provider agencies to engage with parents who are eligible to receive services in a timely manner. This is a critical strategy for retention of those parents who are screened into the study. A web-based notification system was created to provide agencies with the information required to promptly initiate services.

## Conducting a Prevention Trial During Oregon’s Opioid and Methamphetamine Epidemics

This paper identifies the challenges of conducting a substance use prevention study in regions where parental OUD/MUD is an overwhelming burden. By design, hybrid-trial implementation research creates tension between implementation and effectiveness research. There is a balancing act to appropriately test the effectiveness of the prevention intervention while also being responsive to the needs of the study communities and implementing agencies. This trial demonstrates that an early focus on program implementation has provided opportunities to focus on the effectiveness arm of the study. In addition, the trial demonstrates that the study communities initially were more prepared to facilitate parents living with OUD/MUD into the FAIR treatment model, possibly due to the overwhelming need for parental OUD/MUD treatment for families involved with ODHS. Therefore, initially, communities experienced greater challenges with supporting prevention efforts. Thus, if providing the full range of substance use services for families is the goal, it is important to consider readiness for the implementation of both substance use treatment and prevention programs separately, in order to anticipate unique challenges. Indeed, in the current trial, addressing the treatment needs of communities increased bandwidth for identification of prevention referrals.

An early investment in implementation has gradually led to a shift in community focus toward prevention. Being responsive to community needs as an implementation strategy initially presented challenges for recruitment. Yet, over time, the support provided to communities (by implementing both prevention and treatment programs) has alleviated—at least in part—the high need for treatment so that the workforce could begin to consider and refer families in need of prevention. Recently, local leadership across three study counties initiated an internal review of parents who might be eligible and are encouraging CW/SS workers to facilitate referrals—a direct indication that the partnership established with ODHS has promoted increased support for PRE-FAIR. In addition, addressing the full range of need within the study communities has resulted in braided funding opportunities through established partnerships. This includes funding at the state-level that will provide support for services that are essential to the success of the FAIR programs, but often are services that cannot be reimbursed through Medicaid. Thus, from a sustainability standpoint, provider agencies have a greater chance to successfully deliver the FAIR programs over time. Finally, ongoing efforts to enhance social media messaging have led to an increase in eligible self-referrals (Fig. 1B). Thus, efforts to establish and strengthen community partnerships and the ability to evolve recruitment strategies have led to a steady flow of referrals for parents who meet eligibility criteria and could benefit from PRE-FAIR. Identification and recruitment of parents who might benefit from prevention interventions is a challenge nationwide. Previous research has noted challenges related to recruitment of parents (Smokowski et al., 2018), with some noting the need for innovative recruitment strategies that include technology and other strategies to reach parents who otherwise might not access prevention (Edwards-Gaura et al., 2014). Further, it is possible that other communities across the USA could encounter similar barriers during the implementation of OUD/MUD prevention interventions for families that are supported by local CWSs. This is particularly likely as the burden of parental OUD/MUD is relevant across state CWSs. Given the introduction of the Family First Prevention Services Act to states as a funding stream to support family-based substance use preventive interventions, states might have increased motivation to adopt and implement substance use prevention interventions with the national support (USDHHS, n.d.).
